# Identification of Surgeon Burnout via a Single-Item Measure

**DOI:** 10.1093/occmed/kqac116

**Published:** 2022-10-31

**Authors:** J Houdmont, P Daliya, A Adiamah, E Theophilidou, J Hassard, D N Lobo, East Midlands Surgical Academic Network (EMSAN) Burnout Study Group, Jamil Ahmed, Victor Babu, Daryll Baker, David Bartlett, Ian Beckingham, Imran Bhatti, Adam Brooks, Steven Brown, Josh Burke, Hannah Byrne, Ian Chetter, Hannah Cook, James Coulston, Lucinda Cruddas, Richard Dias, Frank Dor, Mukul Dube, Katherine Grant, John Hammond, Rachel Hargest, Theophilus Joachim, Annie Joseph, Naveed Kara, Dimitrios Karavias, Sita Kotecha, Anisa Kushairi, Roshan Lal, Kit Lam, Irwin Lasrado, Rachel Lee, Gurminder Mann, George Mannu, Charles Maxwell-Armstrong, James McCaslin, Frank McDermot, Andrew Miller, Sarah Miller, Jenna Morgan, Sandip Nandhra, Sangara Narayanasamy, Patrick O’Brien, Laura Parry, Kizzie Peters, Marina Pitsika, Emmanouil Psaltis, Kevin Sargen, Panchali Sarmah, Murali Shyamsundar, Chinnappa Reddy, Katie Rollins, Farah Roslan, Joseph Shalhoub, Matt Stanislas, Benjamin Tan, Nilanjana Tewari, Pradeep Thomas, Tony Thomas, Jim Tiernan, Giles Toogood, Karl Trimble, Peter Vauhan, Luke Wheldon, Steven White, Tim White, Imeshi Wijetunga, Michael Wilson, Rebecca Winterborn, Lynda Wyld, Lora Young

**Affiliations:** Centre for Organizational Health and Development, School of Medicine, University of Nottingham, Nottingham, UK; East Midlands Surgical Academic Network, Queen’s Medical Centre, Nottingham, UK; Nottingham Digestive Diseases Centre and National Institute for Health Research (NIHR) Nottingham Biomedical Research Centre, Nottingham University Hospitals and University of Nottingham, Queen’s Medical Centre, Nottingham, UK; East Midlands Surgical Academic Network, Queen’s Medical Centre, Nottingham, UK; Nottingham Digestive Diseases Centre and National Institute for Health Research (NIHR) Nottingham Biomedical Research Centre, Nottingham University Hospitals and University of Nottingham, Queen’s Medical Centre, Nottingham, UK; East Midlands Surgical Academic Network, Queen’s Medical Centre, Nottingham, UK; Nottingham Digestive Diseases Centre and National Institute for Health Research (NIHR) Nottingham Biomedical Research Centre, Nottingham University Hospitals and University of Nottingham, Queen’s Medical Centre, Nottingham, UK; Centre for Organizational Health and Development, School of Medicine, University of Nottingham, Nottingham, UK; East Midlands Surgical Academic Network, Queen’s Medical Centre, Nottingham, UK; Nottingham Digestive Diseases Centre and National Institute for Health Research (NIHR) Nottingham Biomedical Research Centre, Nottingham University Hospitals and University of Nottingham, Queen’s Medical Centre, Nottingham, UK; MRC-Versus Arthritis Centre for Musculoskeletal Ageing Research, School of Life Sciences, University of Nottingham, Queen’s Medical Centre, Nottingham, UK

## Abstract

**Background:**

Burnout is endemic in surgeons in the UK and linked with poor patient safety and quality of care, mental health problems, and workforce sustainability. Mechanisms are required to facilitate the efficient identification of burnout in this population. Multi-item measures of burnout may be unsuitable for this purpose owing to assessment burden, expertise required for analysis, and cost.

**Aims:**

To determine whether surgeons in the UK reporting burnout on the 22-item Maslach Burnout Inventory (MBI) can be reliably identified by a single-item measure of burnout.

**Methods:**

Consultant (*n* = 333) and trainee (*n* = 217) surgeons completed the MBI and a single-item measure of burnout. We applied tests of discriminatory power to assess whether a report of high burnout on the single-item measure correctly classified MBI cases and non-cases.

**Results:**

The single-item measure demonstrated high discriminatory power on the emotional exhaustion burnout domain: the area under the curve was excellent for consultants and trainees (0.86 and 0.80), indicating high sensitivity and specificity. On the depersonalisation domain, discrimination was acceptable for consultants (0.76) and poor for trainees (0.69). In contrast, discrimination was acceptable for trainees (0.71) and poor for consultants (0.62) on the personal accomplishment domain.

**Conclusions:**

A single-item measure of burnout is suitable for the efficient assessment of emotional exhaustion in consultant and trainee surgeons in the UK. Administered regularly, such a measure would facilitate the early identification of at-risk surgeons and swift intervention, as well as the monitoring of group-level temporal trends to inform resource allocation to coincide with peak periods.

## Teaser

Burnout is endemic among surgeons in the UK and has worrying implications for patient safety, surgeons’ mental wellbeing, and workforce sustainability. It is therefore important that mechanisms exist to identify surgeons at risk of burnout. These mechanisms need to be non-intrusive and suitable for regular use. We therefore tested a single-item measure of burnout and established that it could accurately identify consultant surgeons and trainee surgeons at risk of burnout.

**Key learning points**

**What is already known about this subject:**

- Burnout is endemic among surgeons in the United Kingdom (UK).
- Multi-item measures of burnout may be unsuitable for the efficient and early identification of surgeon burnout owing to assessment burden, expertise required for analysis, and cost.
- Brief and single-item measures of burnout might facilitate the identification of burnout in surgeons.

**What this study adds:**

- We assessed whether a report of high burnout on a single-item measure correctly classified cases and non-cases on the ‘gold standard’ Maslach Burnout Inventory.
- The single-item measure demonstrated high discriminatory power on the emotional exhaustion burnout domain, indicating high sensitivity and specificity.

**What impact this may have on practice or policy:**

- A single-item measure of burnout is suitable for the efficient assessment of emotional exhaustion in consultant and trainee surgeons in the UK.
- Administered regularly, such a measure would facilitate the early identification of at-risk surgeons and swift intervention, as well as the monitoring of group-level temporal trends to inform resource allocation to coincide with peak periods.

## Introduction

Burnout is a syndrome resulting from chronic work-related stress characterised by emotional exhaustion, depersonalisation (negativism, cynicism, and increased mental distance from one’s job), and a sense of low personal accomplishment and effectiveness [1]. Burnout is endemic in surgeons in the UK [2] and has implications for patient safety, professionalism and workforce sustainability [3, 4]. An instrument for the assessment of burnout that is brief, non-intrusive and suitable for regular repeated administration would facilitate the early identification of surgeons at risk and swift intervention. It would also support the monitoring of group-level temporal trends to inform resource allocation to coincide with peak periods, while facilitating the evaluation of burnout interventions. Single-item measures, which also require no specialist expertise to analyse and interpret and, unlike some multi-item measures, do not attract payment to the copyright owner, may represent one such instrument. The aim of this study was to determine whether surgeons in the UK reporting burnout on a ‘gold standard’ multi-item measure can be reliably identified by a single-item measure of burnout.

## Methods

Details of sampling and ethics are reported elsewhere [5]. The single-item measure of burnout was developed and validated for use with doctors in the United States [6, 7] and is used extensively in international physician burnout research [8]. The stem question ‘Overall, based on your definition of burnout, how would you rate your level of burnout?’ is followed by five response options: (1) I enjoy my work. I have no symptoms of burnout; (2) Occasionally, I am under stress, and I don’t always have as much energy as I once did, but I don’t feel burned out; (3) I am definitely burning out and have one or more symptoms of burnout, such as physical and emotional exhaustion; (4) The symptoms of burnout that I’m experiencing won’t go away. I think about frustration at work a lot and (5) I feel completely burned out and often wonder if I can go on. I am at the point where I may need some changes or may need to seek some sort of help. Consistent with previous studies, burnout was defined as a response of ≥3.

We also used the ‘gold standard’ 22-item Maslach Burnout Inventory (MBI-HSS (MP)) [9] that is widely used in surgeon research [3]. Threshold scores reflected the upper third of scores in the test authors’ normative sample of physicians and nurses: high emotional exhaustion (≥27), high depersonalisation (≥10), and low personal accomplishment (≤33).

To assess the ability of the single-item measure to discriminate between cases and non-cases on each of the MBI domains we calculated sensitivity, specificity, the proportion of correctly classified cases, and area under the curve (AUC). Sensitivity (true positive rate) was examined by testing the proportion of MBI cases that reported high burnout on the single-item measure. Specificity (true negative rate) was examined by testing the proportion of MBI non-cases that reported no/low burnout on the single-item measure. A test with perfect sensitivity and specificity is indicated by an AUC of 1.0, with discrimination of 0.70-0.79 considered acceptable, 0.80-0.89 excellent, and ≥0.90 outstanding.

## Results

Respondent characteristics have been described previously [5]. Analyses involved respondents who completed both burnout measures and indicated they were a trainee (Core Trainee years 1-2/Specialty Trainee years 3-8) (*n* = 217) or consultant (*n* = 333).

Discrimination statistics are shown in Tables 1 and 2. The AUC on the emotional exhaustion burnout domain was excellent for consultants (0.86) and trainees (0.80). The AUC on the depersonalisation domain was acceptable for consultants and fell short of the acceptability threshold for trainees. On the personal accomplishment domain, the AUC was acceptable for trainees only.

**Table 1.** Single-item burnout measure discrimination for Maslach Burnout Inventory, consultants (*n* = 333)

Burnout domain	Sensitivity (%)	Specificity (%)	Cases correctly classified (%)	AUC (95% CI)
Emotional exhaustion (high)	86	87	86	0.86 (0.82–0.91)
Depersonalization (high)	78	73	74	0.76 (0.69–0.82)
Personal accomplishment (low)	54	70	65	0.62 (0.55–0.69)

**Table 2.** Single-item burnout measure discrimination for Maslach Burnout Inventory, trainees (*n* = 217)

Burnout domain	Sensitivity (%)	Specificity (%)	Cases correctly classified (%)	AUC (95% CI)
Emotional exhaustion (high)	83	76	79	0.80 (0.74–0.86)
Depersonalization (high)	73	64	67	0.69 (0.61to 0.76)
Personal accomplishment (low)	72	69	70	0.71 (0.64–0.78)

## Discussion

In relation to the identification of MBI emotional exhaustion cases and non-cases, a single-item measure of burnout demonstrated excellent discrimination among consultant surgeons and trainee surgeons in the UK.

Strengths of our study include the relatively large sample and assessment of burnout using the ‘gold standard’ MBI. Some features nevertheless need to be considered when interpreting the results. The study was conducted during the COVID-19 pandemic at a time of disruption to surgical activity, yet we have no reason to believe that this would have affected correspondence between the two measures of burnout. Though our surgical trainee sample was sufficient to permit meaningful group-level analyses, it was insufficient to support analyses stratified by stage of training; it is possible that the discriminatory power of the single-item measure differs by stage of training. Questions concerning the resourcing of the regular assessment of burnout in the surgical context require local solutions. Likewise, the availability of personnel to conduct in-depth psychosocial risk assessment with those reporting burnout to identify problematic yet modifiable aspects of work. To identify peak periods and inform timely resource allocation burnout should be assessed at intervals sufficiently regular to be sensitive to changes in demand.

Our findings suggest that a single-item measure of burnout is effective for the assessment of emotional exhaustion among consultant and trainee surgeons in the UK. Leiter and Maslach [10] note that although there is a move in the recent empirical literature towards a simplification of burnout as a one-dimensional construct of exhaustion, burnout remains a three-dimensional construct with the depersonalisation and personal achievement domains being important; work is required to develop brief or single-item measures of these dimensions for surgical application. Involving minimal assessment burden, a single-item measure of burnout is suitable for regular repeated administration within organisational risk assessment activities, allowing for the early identification of at-risk surgeons and swift intervention, as well as the monitoring of group-level temporal trends to inform resource allocation to coincide with peak periods. Such measures could also be used to evaluate burnout intervention efficacy with little disruption to work activity.
